# Epidemiology of methicillin-resistant Staphylococcus aureus (MRSA) in Sweden 2000–2003, increasing incidence and regional differences

**DOI:** 10.1186/1471-2334-6-30

**Published:** 2006-02-21

**Authors:** Mikael Stenhem, Åke Örtqvist, Håkan Ringberg, Leif Larsson, Barbro Olsson-Liljequist, Sara Hæggman, Karl Ekdahl, The Swedish Study Group on MRSA Epidemiology

**Affiliations:** 1 Department of epidemiology, Swedish Institute for Infectious Disease Control, Karolinska Institutet, Solna, Sweden; 2 Department of Medical Epidemiology and Biostatistics, Karolinska Institutet, Solna, Sweden; 3 County Medical Officer for Communicable Disease Control, Stockholm County, Stockholm, Sweden; 4 Deputy County Medical Officer for Communicable Disease Control, Skåne County, Malmö, Sweden; 5 Department of Hospital Hygiene, Sahlgrenska University Hospital, Göteborg, Sweden; 6 Department of bacteriology, Swedish Institute for Infectious Disease Control, Solna, Sweden; 7 European Centre for Disease Prevention and Control (ECDC), Solna, Sweden

## Abstract

**Background:**

The occurrence of methicillin-resistant *Staphylococcus *aureus (MRSA) has gradually become more frequent in most countries of the world. Sweden has remained one of few exceptions to the high occurrence of MRSA in many other countries. During the late 1990s, Sweden experienced a large health-care associated outbreak which with resolute efforts was overcome. Subsequently, MRSA was made a notifiable diagnosis in Sweden in 2000.

**Methods:**

From the start of being a notifiable disease in January 2000, the Swedish Institute for Infectious Disease Control (SMI) initiated an active surveillance of MRSA.

**Results:**

The number of reported MRSA-cases in Sweden increased from 325 cases in 2000 to 544 in 2003, corresponding to an overall increase in incidence from 3.7 to 6.1 per 100000 inhabitants. Twenty five per cent of the cases were infected abroad. The domestic cases were predominantly found through cultures taken on clinical indication and the cases infected abroad through screening. There were considerable regional differences in MRSA-incidence and age-distribution of cases.

**Conclusion:**

The MRSA incidence in Sweden increased over the years 2000–2003. Sweden now poises on the rim of the same development that was seen in the United Kingdom some ten years ago. A quarter of the cases were infected abroad, reflecting that international transmission is now increasingly important in a low-endemic setting. To remain in this favourable situation, stepped up measures will be needed, to identify imported cases, to control domestic outbreaks and to prevent transmission within the health-care sector.

## Background

Since its discovery in 1961, the occurrence of methicillin-resistant *Staphylococcus *aureus (MRSA) has gradually become more frequent over the world [1-3]. In the early 1990s, United Kingdom, the Netherlands and the Nordic countries were essentially non-endemic for MRSA. Since then, MRSA has become established as a common nosocomial pathogen in the British health-care system [4,5]. The Netherlands and the Nordic countries still have favourable MRSA-situations, but are now observing steadily increasing numbers of new cases reported [6-9]. During the late 1990s, Sweden experienced a large health-care associated outbreak in the Västra Götaland County, which with resolute efforts were overcome [10,11]. Subsequently, MRSA carriage and disease was made notifiable from January 1, 2000, according to the Swedish Communicable Disease Act.

The aim of the present study was to investigate the epidemiology in a low- or non-endemic situation, as is still the case in Sweden, which in turn may reveal basic mechanisms for MRSA spread that may not be perceived in countries where MRSA is more or less ubiquitous.

## Methods

Under the Communicable Disease Act, both the microbiological laboratory having diagnosed MRSA and the clinician having seen the patient with the pathogen are obliged to report all new cases of either colonisation or infection with MRSA to the County Medical Officer for Communicable Disease Control and to the Swedish Institute for Infectious Disease Control (SMI). At the SMI, the notifications from the two sources are merged into case records, using a unique personal identification number issued to all residents in Sweden, and used in all health care contacts. From the start of MRSA being notifiable in January 2000, the SMI had in place an active surveillance system feeding detailed information on all cases of MRSA into a national database. The SMI actively completed and validated the epidemiological information on each notified case, through the courtesy of specially appointed contact persons in each county, with data compiled after the epidemiological investigation around the case was completed. Cases were found through cultures taken on clinical indication as well as through screening and contact tracing. Each county has its own rules regarding screening and contact tracing practices, these practices are however in reality similar. Sites cultured include vestibulum nasi, perineum, breakages in the skin barrier, such as wounds, eczemas, etc., and urinary catheters. The oropharynx is also commonly cultured for screening and contact tracing purposes. Patients having received health care outside of Sweden, or within Sweden in areas where MRSA prevalence is known to be high, are screened for MRSA in all counties. In this study we used surveillance data from January 2000 to December 2003. Isolates from all new cases were sent to the SMI and the laboratory diagnosis of MRSA was confirmed with polymerase chain reaction (PCR) for the *mecA *and the *nuc *genes [12]. Demographic data were obtained from the official statistics published by Statistics Sweden (SCB) [13]. Through a questionnaire to all 29 clinical microbiology laboratories in Sweden, denominator data on the number of individuals with cultures positive for *Staphylococcus aureus *were requested by the SMI.

Within the present study a case of MRSA was defined as a case of either colonisation or infection with MRSA. When a case is said to be "infected" with MRSA, this refers to the transmission of MRSA to this person, with resulting colonisation or infection. The MRSA incidence was defined as the number of new cases, during a specified year and in a specified geographic area, divided by the population number of the same area in the same year and expressed as number of cases per 100000 inhabitants. The MRSA proportion was defined as the number of new cases, during a specified year and in a specified geographic area, divided by all cases with a laboratory diagnosis of *Staphylococcus aureus*, expressed as a percentage.

**Figure 1:**
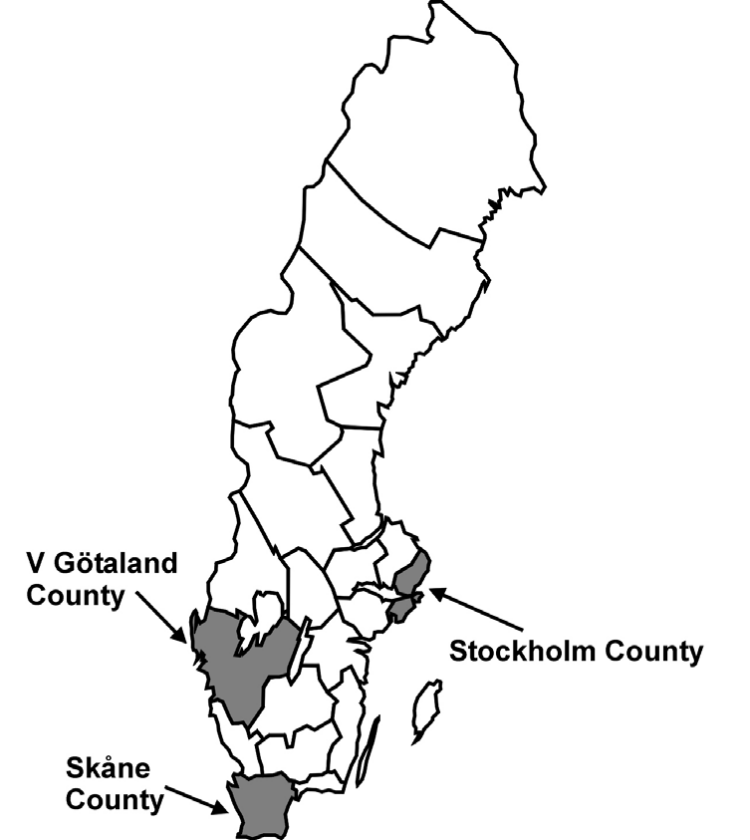
Map of Sweden showing county boundaries and the three big city counties: Skåne, Stockholm and Västra Götaland.

Sweden is divided in 21 counties. Three of these, Stockholm County, Västra Götaland County and Skåne County, have considerably more inhabitants than the rest, 1.8, 1.5 and 1.1 million inhabitants, respectively, and also include the three biggest cities, Stockholm, Göteborg, and Malmö (Figure 1). The other counties have between 57000 and 414000 inhabitants (average of 247000). Since the three most populous counties comprised 71% of the MRSA cases, but only 50% of the total Swedish population, they were studied separately. The other 18 counties had considerably lower MRSA-incidence, sometimes with just a very small number or no cases at all in single years, and were for that reason analysed together.

Correlation analysis was performed in Stata 8.2 [14].

## Results

**Figure 2:**
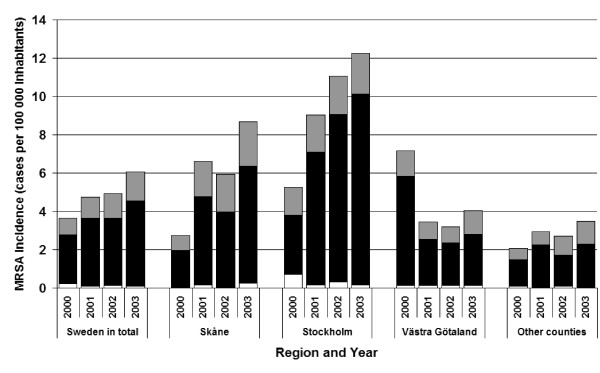
Incidence (cases per 100000 inhabitants) of MRSA cases, infected in Sweden (■), abroad () and unknown where infected (□), notified in Sweden in total, in each of the counties Skåne, Stockholm, Västra Götaland and the other 18 counties presented together, for the years 2000–2003.

The number of reported MRSA cases in Sweden increased from 325 cases in 2000 to 423 in 2001, 441 in 2002 and 544 in 2003. A total of 1,733 cases were thus reported during the entire study period, 1,240 infected in Sweden (domestic cases) and 443 infected abroad. As shown in Figure 2 these numbers corresponded to an overall increase in incidence from 3.7 to 6.1 per 100,000 inhabitants. The total increase was due to a combined increase in domestic cases (from 2.5 to 4.4 per 100,000) and in cases infected abroad (from 0.92 to 1.6 per 100,000). The proportion of cases infected abroad was constantly near 25% of all reported cases, for the whole of the country.

The Skåne County and the Stockholm County contributed most to the overall increase in MRSA during the period (Figure 2). Contrary to this, the incidence of domestic cases in the Västra Götaland County was higher in 2000 than later during the study period, reflecting the aftermaths of the large outbreak in that county peaking in 1998 [10,11]. In the three large counties, changes in the number of cases infected domestically and abroad paralleled each other. In the smaller counties a steadily increasing incidence of cases from abroad was seen. In Stockholm the increase in domestic incidence was so pronounced, compared to the increase in cases infected abroad, that the proportion of cases infected abroad decreased from 28% in 2000 to 17.5% in 2003.

Cultures positive for MRSA were obtained: 1) on clinical indications (e.g. symptomatic patients), 2) as screening samples from patients belonging to a risk-group (e.g. patients having received health-care abroad), or 3) as part of the contact-tracing in the investigation around an already identified case. The domestic cases were predominantly found through cultures on clinical indications (58%), with contact tracing as the second most common indication (35%), and screening on the third place (5%); in the remaining 1% the reason for culture was unknown. The patients infected abroad were mostly found through screening (53%), followed by clinical indication (39%) and contact tracing (8%).

**Figure 3:**
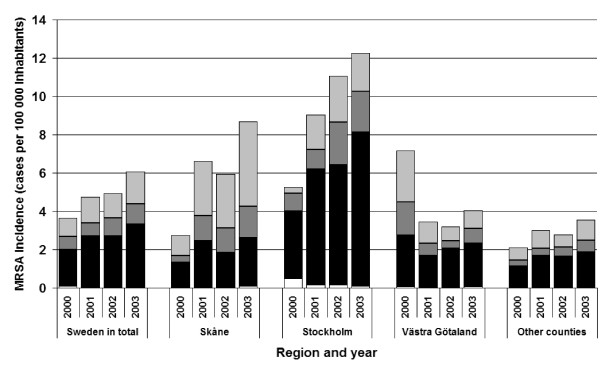
Incidence (cases per 100000 inhabitants) of different indications for culture: clinical indication (■), screening (), contact tracing () and unknown/other indication (□), notified in Sweden in total, each of the counties Skåne, Stockholm, Västra Götaland and the other 18 counties presented together for the years 2000–2003.

Figure 3 shows that the incidence of MRSA cases discovered by cultures taken on clinical indication varied around two cases per 100000 inhabitants, without a clear trend over time, for all parts of the country apart from the Stockholm County, where the incidence of clinical cases rose year by year from 3.5, in 2000, to 8.0 cases per 100000 inhabitants in 2003. The Skåne County had the largest proportion of cases found through either contact tracing or screening (range 52% to 70%), compared to the Västra Götaland County (range 35% to 62%), the Stockholm County (range 24% to 42%) and the smaller counties (range 41% to 47%).

**Figure 4:**
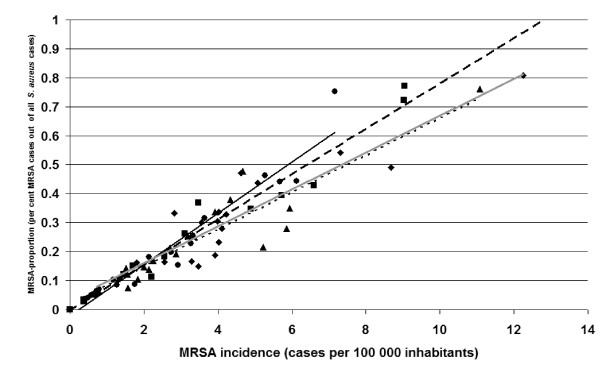
Scatter-plot and linear curve-fit of MRSA-proportion (percentage of MRSA-cases out of all *Staphylococcus aureus *cases) against MRSA-incidence (cases per 100000 inhabitants) for the years 2000 (● and ; N = 19), 2001 (■ and ---; N = 16), 2002 (▲ and .....; N = 19) and 2003 (◆ and ; N = 20). Each data point represents one county and one or several laboratories within this county.

Figure 4 shows a scatter plot of the proportion of MRSA-cases out of all cases diagnosed with *S. aureus *against incidence of MRSA (MRSA cases per 100,000 inhabitants) for each of the Swedish counties and each of the years 2000 – 2003. The two measures were highly correlated to each other – Spearman correlation coefficients (r_Spearman_) 0.98, 0.99, 0.92 and 0.90, respectively for each of the years 2000–2003 (p < 0.0001 for all of the years).

**Figure 5:**
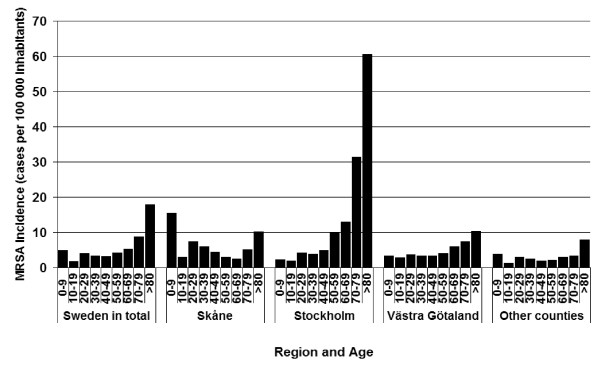
Average yearly incidence of MRSA per age group (cases per 100000 inhabitants in each of the ten-year age-groups from 0–9 years to 80 years and above) and county, in Sweden, over the years 2000 to 2003.

The age distribution of cases showed marked differences between counties, as seen in Figure 5. Stockholm, Västra Götaland and the smaller counties had their highest incidence in the age-group above 80 years of age, with 61, 10 and 8.0 cases per 100,000 inhabitants respectively. Skåne on the other hand had the highest age related incidence in the 0–9 years group, with 15 cases per 100,000 inhabitants, and in the ages above 80 years the incidence was 10 cases per 100,000 inhabitants. Overall, MRSA incidence was lowest in the ages 10–19 years.

The average incidence of MRSA acquired abroad, over the period 2000 – 2003, was highest in the age group 0–9 years (1.8 cases per 100,000 inhabitants and year) and in the age group 20–29 years (1.9 cases per 100,000 inhabitants and year), in Sweden in total. The average incidence of domestically infected cases increased markedly with age, from 2.1 cases per 100,000 inhabitants in the age group 40–49 years to 16.5 cases per 100,000 inhabitants in the age group above 80 years, while the incidence of cases infected abroad decreased with age.

## Discussion

This study shows a year-by-year increasing incidence of MRSA, in Sweden, since this pathogen became notifiable in 2000. There were also notable regional differences between counties both in total and age-specific incidence. The three big city regions had higher incidences than the rest of the country. The incidence in the Västra Götaland County in 2000 represents the tail of a health-care related outbreak in that county in the late 1990s. Since the beginning of our study period in 2000, the Stockholm County has experienced several simultaneous health-care associated outbreaks, which were reflected in the incidence of 10 domestic cases per 100,000 inhabitants. These outbreaks could explain the high incidences in the oldest age groups noted in the Stockholm County. The Skåne County, the other big city region with an increasing incidence during the study period, had a totally different age profile for the MRSA-cases reported, reflecting fewer health-care related outbreaks and a higher proportion of community-acquired MRSA.

This is the first nation-wide study on the MRSA-situation in Sweden, one of few countries with very low MRSA endemicity. Finland and the Netherlands have recently reported on their respective MRSA-epidemiology [7,8]. From Norway official surveillance statistics are available [9]. The occurrence of MRSA is declared to be increasing in all of these countries, but still on a lower level than most other countries [15].

The present study was a population-based study starting from a mandatory notification system. The Swedish statutory notification system was recently assessed by Jansson et al. [16]. It was concluded that it is a highly sensitive reporting system, further improved by the double reporting of clinicians and laboratories. The overall sensitivity was found to lie well above 90% for the studied diagnoses.

MRSA was first observed as an increasing problem within health care institutions. In recent years more and more attention has been paid to MRSA outside health care, designated community-associated MRSA (CA-MRSA). Consequently, most studies have focused either on spread of MRSA as an infection control problem within hospitals and other health care institutions, on the epidemiology of CA-MRSA, or approached MRSA from a microbiological point of view. However, to get a more complete picture of MRSA-epidemiology such studies need to be complemented by regional and national population based studies, approaching MRSA occurrence as a public health issue in society as a whole.

A quarter of the Swedish MRSA-cases in 2000–2003 were infected abroad, as shown in this study. These cases stand out more distinctly against the low-endemic Swedish MRSA-situation, than elsewhere. For the same reason imported MRSA-cases are, with few exceptions [8,9], rarely accounted for in studies and national statistics. MRSA in low-endemic circumstances, however, constitute opportunities to study international transmission of MRSA. Even though the overall proportion of MRSA acquired abroad was constant over time, this was the combined result of two opposing trends. In the Stockholm and the Skåne Counties increasing incidences due to domestic outbreaks and community transmission with fairly constant incidence of imported cases were noted, while in the Västra Götaland Region (from 2001) and the other counties, the incidence was increasing more in the imported than in the domestic cases.

Clinical indication was a common motive for the culture that identified the MRSA-cases infected both domestically (59%) and abroad (39%). It was, however, reassuring, regarding the way the counties operate their MRSA situations, to see that the majority of cases infected abroad were found through screening practices and as much as a third of the domestically infected cases were found by contact-tracing. There were no trends for these parameters over the study period, in the country as a whole.

Regional differences within countries have been observed by other authors [1,5,17-20]. Activity in screening, outbreak investigations and contact tracing regarding MRSA may differ between regions and countries. Reporting of numbers of MRSA-cases including cases first identified through cultures taken for screening or outbreak-investigation purposes may thus be subject to bias. For this reason information was collected, and accounted for in this study, on each case concerning the indication for the culture that first identified the individual as a MRSA-case. In the present study the Skåne County was shown to have the second highest total incidences of MRSA during the years 2001–2003. The majority of cases in Skåne (between 63 and 70 per cent) where, however, found through active search for MRSA. This illustrates how misleading a comparison between regions can be if only total incidences had been used. If the number of clinically found MRSA cases is assumed to reflect the occurrence of MRSA more truly, the Skåne County did not have a higher occurrence of MRSA than the rest of Sweden.

The MRSA proportion has been described as a rough measure of MRSA occurrence [18]. We had the opportunity to correlate the MRSA proportion to incidence figures based on census data. In the Swedish setting and during the time period studied these two ways of measuring MRSA occurrence correlated excellently.

The occurrence of MRSA is frequently measured as the proportion of MRSA out of all *S. aureus*. One reason for using this proportion is convenience. Any bacteriologic laboratory can easily summate cultures positive for *S. aureus*, and so get the denominator for the proportion. Another reason for using this proportion may be that population denominators are difficult to estimate if the catchment population of a laboratory is not clearly defined. The validity and comparability of all measures of occurrence depend on the case definition and the case detection practices used. However, the MRSA proportion is a relative measure of occurrence depending not only on the amount of MRSA, but also on the total amount of *S. aureus*. Circumstances could thus affect the proportion in a less predictable way than ordinary population based measures of incidence and prevalence.

We interpret our finding of high correlation between MRSA proportion and MRSA incidence in this study as basically a correlation between denominators – the total number of cultures positive for *S. aureus *per county and the population of the same county. The correlation suggests that the overall culturing practices were similar between Swedish counties, since the yield of *S. aureus *was similar in relation to population. The finding shows that in the Swedish setting, with low MRSA occurrence, and during the period studied, these two ways of measuring MRSA occurrence could be used interchangeably. However, the correlation in this study between MRSA proportion and incidence should not be interpreted as evidence that the proportion of MRSA out of all *S. aureus *is generally a valid estimate of MRSA occurrence in the population.

## Conclusion

This study has shown that the MRSA incidence in Sweden increased over the years 2000–2003. Sweden now poises on the rim of the same development that was seen in the United Kingdom some ten years ago. A quarter of the cases were infected abroad, reflecting that international transmission is now increasingly important in a low-endemic setting. To remain in the present favourable situation, stepped up measures will be needed, to identify imported cases, to control domestic outbreaks and to prevent transmission within the health-care sector. MRSA-proportion and MRSA-incidence correlated excellently as measures of MRSA-occurrence. Further and more detailed studies of the domestic Swedish MRSA epidemiology are needed as well as studies of the consequences of international transmission of MRSA in low-endemic settings.

## Competing interests

The author(s) declare that they have no competing interests.

## Authors' contributions

MS collected, completed and validated the data on the national level, analysed the data and drafted the article. ÅÖ provided the data from Stockholm and contributed to the drafting of the the article. HR provided the data from Skåne and critically revised the article. LL provided the data from Västra Götaland and contributed to the drafting of the article. BOL and SH were responsible for bacteriological diagnostics on the national level and contributed to the drafting of the article. KE was supervisor of MS and took part in the analysis of the data and drafting of the article.

## Pre-publication history

The pre-publication history for this paper can be accessed here:


